# Knowledge, Attitudes, and Perceptions of Medical and Paramedical Students Toward Human Milk Banks and Breast Milk Donation

**DOI:** 10.7759/cureus.55145

**Published:** 2024-02-28

**Authors:** Anugya A Taksande, Sachin Tote, Amar Taksande, Chaitanya Kumar Javvaji

**Affiliations:** 1 Pediatrics, Jawaharlal Nehru Medical College, Datta Meghe Institute of Higher Education and Research, Wardha, IND; 2 Anatomy, Jawaharlal Nehru Medical College, Datta Meghe Institute of Higher Education and Research, Wardha, IND

**Keywords:** breastfeeding, food and nutrition, newborn and child health, human milk bank, breast milk

## Abstract

Background and objectives

Human milk benefits for both mothers and babies are widely acknowledged. Human milk banks (HMBs) are essential in providing newborns who would not otherwise have access to pasteurized and stored human milk. The objective of this research was to investigate the understanding, opinions, and outlooks of medical and paramedical students regarding breast milk donation and the concept of an HMB.

Material and methods

A total of 398 professional female students specializing in physiotherapy, nursing, and medicine were given an online self-administered questionnaire as part of a descriptive cross-sectional study. Data on the characteristics, HMB knowledge, awareness levels, and attitudes toward HMB and breast milk donation were to be gathered through the questionnaire. Every student's answer to the survey was kept private and confidential.

Result

According to the survey, 188 students (47.24%) belong to the medical college, 126 students (31.66%) belong to the physiotherapy college, and the remaining 84 students (21.11%) are from the nursing college. A total of 294 students (74.12%) had heard about human milk banking before and received information from health professionals (195 students, 48.99%), the news (67 students, 16.83%), and family and friends (61 students, 15.33%). However, only 224 students (56.28%) are willing to feed their babies with HMB milk in the future. On the other hand, 216 students (54.27%) are ready to donate breast milk to HMB. The majority of students have a favorable opinion of breast milk donation. A staggering 394 students (98.99%) think that donating human milk can save babies. Furthermore, 379 students (95.23%) think that all the nutrients needed for a baby's healthy development are found in human milk. Merely 350 students (87.93%) think that breastfeeding does not result in malnourishment for either the mother or the baby. When there is an excess of milk, the majority of students (378 or 94.97%) think that there is nothing wrong with donating it. Moreover, 312 (80.41%) students are happy to donate milk in the future. A total of 373 students (93.71%) are overjoyed that a sick baby will survive because of their donated milk. Only 100 students (25.12%) will give milk, though, and only to the infants of their friends and relatives, never to complete strangers.

Conclusion

A dearth of knowledge pertaining to human milk banking is rampant among medical and paramedical female students, yet the gravity of the circumstances remains concealed. Consequently, it is imperative to extensively educate all Indian communities about the concept of human milk banking to gain widespread acceptance. This research sheds light on the issue and promotes scientific knowledge of HMB, as many students are unaware of it.

## Introduction

Continuing breastfeeding until the child reaches at least two years of age is vital for their development and growth, especially during the first six months of life. Breast milk's unique composition provides abundant nutrition, immunological protection, and sufficient hydration for babies [1]. These factors significantly reduce the occurrence of illness and death among newborns. The advantages of breast milk are widely recognized, and no replacement can match them. Breast milk includes vital minerals, digestive enzymes, hormones, and vitamins in addition to the right proportions of proteins, lipids, and carbohydrates. A baby's defense against infections is further aided by the antibodies and the mother's lymphocytes in breast milk. Breast milk provided by a woman other than the child's mother is referred to as donor human breast milk. This milk is given to needy babies after being processed at a milk bank [2-3]. When a mother is not able to produce a sufficient amount of milk or if breastfeeding is contraindicated, the recommended alternative is to use donated breast milk for her infant.

Nevertheless, preserved milk can be a suitable substitute when obtaining a mother's milk is impractical. Regrettably, there is opposition to breastfeeding among certain lactating mothers. Breast milk from donors is an invaluable asset for premature and high-risk infants, protecting them from conditions such as necrotizing enterocolitis and sepsis. While the well-established benefits and importance of breastfeeding for both mothers and infants are widely recognized, the act of donating breast milk remains relatively uncommon in our society [4]. In addition to addressing many issues, breast milk donation helps reduce hospital stays. The success of any new healthcare intervention depends significantly on its acceptability. The increasing number of premature babies worldwide, particularly in low-income countries, presents a challenge for neonatologists and breast milk banks to ensure adequate nutrition for infant development. Because of safety apprehensions, numerous mothers exhibit reluctance to utilize donor milk. Before instituting a new milk bank, it is essential to gauge the demand and availability of milk donors [5]. The primary aim of this research is to assess the understanding, attitudes, and perspectives of medical and paramedical students regarding human milk banks (HMBs) and breast milk donation.

## Materials and methods

After obtaining approval from the Datta Meghe Institute of Higher Education and Research (DMIHER) Institutional Review Board (IRB) under approval number 107-2021, a cross-sectional study was conducted at Jawaharlal Nehru Medical College, DMIHER, Sawangi (Meghe), Wardha. The study was carried out over two months, starting in September 2023. The inclusion criteria necessitated a minimum age of 18 years and enrollment in the medical, nursing, or physiotherapy college for female students only. The exclusion criterion was non-consent to participate in the study. For the medical, nursing, and physiotherapy students, a questionnaire was distributed in the form of Google Docs.

The participants were then asked to fill out a carefully crafted questionnaire designed to align with the study's goals. This survey sought to collect information on participants' characteristics, their understanding of HMBs, and their attitudes and perceptions regarding both donor breast milk and HMBs. The response of each student to the questionnaire was kept completely confidential. The English-based questionnaire used was formulated following an extensive review of the literature. The questionnaire was administered in English language only. Given that the study participants were students attending English-medium educational institutions, there was no need for translation. To assess the questionnaire's validity, clarity, and length, a pilot study was conducted involving 50 student participants. The knowledge and attitude of HMB were assessed using seven questions. Finally, perceptions toward HMB and DBM were evaluated using a 13-item questionnaire, where the “agree” answers were coded as 1 and “disagree” answers as 0.

The sample size required for the study was determined through the utilization of the Epidemiological Information Package (EPI) Info Version 7.2, developed by the Centers for Disease Control and Prevention (CDC) in Georgia, USA. The software indicated that a minimum of 384 participants were needed based on a margin of error of ±5%, a confidence level of 95%, and an expected frequency of 50%. After the completion of the survey, all the student participants were given knowledge of the HMB, and the process of functioning of the HMB and its benefits for the babies were explained in detail.

Statistical analysis

Descriptive statistics were employed to summarize the characteristics of the entire student group. The demographic characteristics of the participating students were expressed using frequency and percentage distribution. Continuous variables were represented using the mean. The differences in knowledge and attitudes among participants with varying characteristics were not assessed. All data analyses were carried out using STATA Version 10.0 (StataCorp LLC, College Station, Texas, USA).

## Results

The characteristics of female students participating in the study are shown in Table 1. Only female students were included in the study. Results showed that 95% of the participants belong to the age group of 20 years, 47.24% of students are from medical college, 31.66% of students are from physiotherapy college, and the remaining 21.11% are from nursing college. The majority of students (72.61%) belonged to the Hindu religion, followed by the Buddhist religion (19.60%) (Table 1).

**Table 1:** The characteristics of students participating in the study

Characteristics of female students	Number (n = 398)	Percentage (%)
Student		
Medical student	188	47.24
Nursing student	84	21.11
Physiotherapy student	126	31.66
Religion		
Hindu	289	72.61
Buddhism	78	19.6
Muslim	17	4.27
Jainism	12	3.02
Christian	2	0.5

Knowledge and attitudes of students about HMB and breast milk donation

According to the findings, 74.12% of students had heard of human milk banking before and received information from health professionals (195, 48.99%), the news (67, 16.83%), family and friends (61, 15.33%), etc., while 25.88% of students did not know about it. When considering the future feeding practices of the students, it was observed that only 43.72% were inclined to nourish infants with human milk banking milk. On the other hand, the remaining 56.28% expressed reluctance toward this approach. Among those who were unwilling, 27.39% cited concerns about disease transmission, while 17.84% held the belief that breast milk from other women is nutritionally inferior to that of one's lactating mother. Regarding the willingness to donate breast milk to HMB, a majority of 54.27% of the students expressed their openness to contributing. However, a significant proportion of 45.73% did not share this sentiment. Among the reasons for not wanting to donate, 20.35% believed it could lead to substantial health issues, 11.31% felt their baby required more breast milk, 29.65% simply disliked the idea, and 2.51% were restricted by religious or cultural reasons. Furthermore, the findings revealed that a considerable number of students, specifically 43.97%, expressed interest in joining the HMB association and providing financial support. Additionally, 36.18% of students displayed interest in advancing their professional development toward leadership roles. These findings are summarized in (Table 2).

**Table 2:** Students' knowledge and attitudes on human milk banking and donor human milk (n = 398) HMB, human milk bank

Knowledge and attitude toward human milk banking	Number (n = 398)	Percentage (%)
1. Have you ever heard about HMB		
Yes	295	74.12
No	103	25.88
2. Source of knowledge of HMB		
Health professional	195	48.99
News	67	16.83
Family and friends	61	15.33
Social media	9	2.26
Television	7	1.76
Internet	5	1.26
3. In the future, are you willing to feed the baby with milk from HMB?		
Yes	224	56.28
No	174	43.72
4. If not, what is your reason for not wanting to use milk from HMB?		
Fear of disease transfer	109	27.39
Culture or religion prohibits you	71	17.84
Breast milk from another woman is nutritionally less	19	4.77
Others	10	2.51
5. In the future, will you volunteer to donate your breast milk to HMB?		
Yes	216	54.27
No	182	45.73
6. If your answer is no, what is your concern for not donating		
Make great health problems for you	81	20.35
Affect your physical appearance	16	4.02
Your baby needs more	45	11.31
Your religion or culture prohibits you	10	2.51
Dislike	118	29.65
7. Will you volunteer to be a member of the HMB association?		
Financial support	175	43.97
Leader professional supports	144	36.18
Advocacy	69	17.34
Others	02	0.5

Perceptions of students about HMB and breast milk donation

A total of 394 students (98.99%) think that babies can be saved by human milk donation. Three hundred and seventy-nine students (95.23%) think that human milk has all the nutrients needed for a baby to grow and thrive. According to 380 students (95.47%), breast milk donation diminishes breast engorgement, which proves beneficial for the well-being of mothers. Merely 350 students (87.93%) are inclined toward the idea that the act of donating breast milk does not lead to malnourishment of the mother or her baby. The majority of students, accounting for 378 students (94.97%), are of the view that there is no harm in donating milk if there is an excess production of it. On the other hand, 193 students (48.49%) believe that their baby should solely consume their milk and not any other, while 205 students (51.51%) do not concur with this statement. Similarly, 155 students (38.94%) are in favor of restricting the consumption of their milk to their baby and not others, but 243 students (61.06%) disagree with this notion. In the context of this study, a majority of 312 students (78.39%) express their willingness to donate milk in the future, whereas 86 students (21.61%) are not inclined to do so. Moreover, 353 students (88.69%) think that religion, caste, or culture have little bearing on milk donation. In addition, 380 students (95.47%) feel a sense of joy and contentment knowing that their donated milk will be instrumental in the survival of a sick baby. However, a mere 100 students (25.12%) would confine their milk donation only to the babies of their family members and friends, excluding any strangers. Finally, 140 students (35.17%) express concern that their milk might not be enough for their own child if they gave it to other newborns (Table 3).

**Table 3:** Students' perceptions of HMB and DHM DHM, donor human milk; HMB, human milk bank; MS, medical student; NS, nursing student; PS, physiotherapy student; Q. no., question number

Q. no.	Question on students' perception of HMB and DHM	MS (n = 188)	NS (n = 84)	PS (n = 126)
Q1	I believe that human milk donation can save babies.			
	Agree	188	84	122
	Disagree	00	00	04
Q2	I believe that human milk contains all the essential nutrients required for the healthy existence and complete development of the baby.			
	Agree	178	84	117
	Disagree	10	00	09
Q3	I believe that donating breast milk is good for a mother’s health, too, as it reduces breast engorgement.			
	Agree	182	80	118
	Disagree	06	04	08
Q4	I believe that donating breast milk does not cause malnutrition in the mother or her baby.			
	Agree	174	78	98
	Disagree	14	06	28
Q5	I believe that there is nothing wrong with donating milk in case of excess production of milk.			
	Agree	182	76	120
	Disagree	02	08	06
Q6	I believe that my baby should get only my milk and not others.			
	Agree	60	48	85
	Disagree	128	38	41
Q7	I believe that my milk should be given only to my baby and not to others.			
	Agree	35	44	76
	Disagree	153	40	50
Q8	I am interested in donating milk in the future.			
	Agree	140	70	102
	Disagree	48	14	24
Q9	I believe that culture/caste/religion does not have any role in milk donation.			
	Agree	168	76	109
	Disagree	20	08	17
Q10	I believe that it is good to donate milk to babies who are struggling to survive.			
	Agree	178	80	122
		Disagree	10	04
Q11	I feel very happy that my donated milk will be the reason for the survival of a sick baby.			
	Agree	177	78	118
	Disagree	11	06	08
Q12	I will donate milk only to my family members and friends’ babies and not to any strangers.			
	Agree	52	28	20
	Disagree	136	56	106
Q13	I believe that if I am giving my milk to other babies, then it may not be sufficient for my baby.			
	Agree	42	44	54
	Disagree	146	40	72

## Discussion

Breastfeeding is regarded as the esteemed standard that encompasses all essential requirements for newborns, even those who are unwell. Human breast milk offers unique advantages for newborns, evident in both short-term and long-term health outcomes for both the mother and child [6-8]. The first HMB was established in Vienna, Austria, in 1909. This pioneering concept gained swift acceptance in numerous locations worldwide, leading to the establishment of HMBs across the globe. India's inaugural HMB was founded at Sion Hospital in 1989, serving approximately 3,000 to 5,000 infants annually with essential services. As human milk is specific to the species, it possesses a comparative superiority over alternative formulas for newborn feeding. The collection, screening, pasteurization, and distribution of donated milk to recipients are all carried out in accordance with well-defined guidelines [9-10]. The utilization of donor milk varies across different regions of the world. The global community's dedication to reducing newborn mortality and promoting child survival has resulted in a swift expansion of HMB facilities. Integrating the HMB system with existing hospitals can enhance donor convenience and reduce barriers to donation. Collaborating with community leaders to raise awareness about healthcare is instrumental in supporting donor recruitment. Regrettably, due to a lack of awareness within the community, breast milk is often discarded without consideration for donation when an infant passes away. In hospital settings, when a mother's milk is unavailable, it is recommended to provide pasteurized human donor breast milk to sick newborns instead of commercial formula. Despite advancements in baby formulas, human breast milk remains the most effective source of nutrition. HMBs play a crucial role in ensuring that infants receive this essential nutrition. Offering human milk is particularly beneficial in reducing the risk of necrotizing enterocolitis and sepsis in premature infants.

HMBs serve as carriers for various elements such as drugs, genes, immunoglobulins, and vaccines, contributing to the optimal growth and maturation of infants [7-9]. Breast milk donations can serve as a temporary solution to meet the nutritional needs of newborns when their mothers' milk supply is insufficient or when the baby is too young to nurse. It is essential for medical and paramedical students to expand their knowledge and foster a positive attitude toward breastfeeding, supporting breastfeeding mothers and advocating for the importance of HMBs in the future. Nursing students play a significant role in raising public awareness about the establishment of breast milk banks. Additionally, medical students should receive more instruction or training on breastfeeding, breast milk donation, and the establishment of breast milk banks. While awareness and acceptance of HMBs may be low, a considerable number of students have shown a positive attitude toward human donor milk. Educational campaigns are necessary to promote HMBs. Table 4 presents the results of various studies conducted on HMBs and donor human milk.

**Table 4:** Studies on human milk bank and donor human milk DHM, donor human milk; HMB, human milk bank

Author (year of publication)	Study design	Aim	Sample size	Participants	HMB/ DHM	Result	Conclusion
Mackenzie C et al. (2013) [11]	In-depth semi-structured interviews	This study explored mothers' knowledge of and attitudes toward HMBs	12	Mothers who were breastfeeding and/or had preterm or sick babies	HMB and DHM	Considering that it would be convenient (particularly if dropping off milk) and not unduly time-consuming, breastfeeding moms who were considering becoming donors all agreed to donate their breast milk to an HMB.	Study participants would welcome having access to an HMB for both donating and receiving milk in South Australia.
Karadag A et al. (2015) [12]	Descriptive cross-sectional study	To ascertain mothers' knowledge, attitudes, and opinions about HMBs, breast milk, wet nursing, milk kinship, and newborn feeding	1,042	Mothers who delivered	HMB	Approximately 42.4% of mothers were against establishing HMBs in Turkey. Only 9.2% of mothers would donate their breast milk to Western-style HMBs.	The majority of mothers in this study oppose Western-style HMBs but are more positive about an alternative HMB when religious concerns are addressed.
Zhang N et al. (2020) [13]	Cross-sectional survey	To evaluate mothers' understanding of HMBs, their receptiveness to human donor milk (HDM), and their willingness to donate and/or utilize HDM, and to identify the factors influencing milk donation and/or the utilization of HDM	1,048	Mothers who returned to the hospital for postpartum follow-up within six months	HMB and DHM	20% of the participants were previously aware of HMBs and milk donation. The highest accuracy in responses was observed regarding the benefits of breast milk, whereas the lowest accuracy was related to the acceptance of donor human milk. A majority, 75.3%, expressed support for the establishment of HMBs, while 81.3% were in favor of donating breast milk, and 38.3% supported the acceptance of donor human milk. There was limited interest among participants in donating breast milk and disseminating knowledge about breast milk banks, with only 28.3% indicating continuous donation.	A majority of postpartum women support HMBs and are more willing to donate breast milk than receive donor milk. Lack of knowledge and safety concerns hinder women from donating or accepting donor milk.
Aishwarya K et al. (2021) [14]	Cross-sectional study	To assess maternal knowledge of HMBs, acceptability of HDM, willingness to donate and/or use HDM, and determinants of milk donation and/or use of HDM	419	Women	HMB and DHM	14.79% were aware of HMBs. In India, 10.3% are aware of HMBs. 74.22% are prepared to donate milk. 61.57% are open to using HDM. The main motivation for donating (72.55%) was altruism. Fear of not having enough milk (39.13%) was the main obstacle. Women favored giving to needy infants (68.17%) and family members (73.31%) over HMBs (45.02%). Compared to HMBs (54.26%), women preferred to receive milk from relatives (82.95%). The primary obstacles to the adoption of HDM were reluctance (37.37%) and formula convenience (37.37%). The formula was chosen over HDM by women at private universities (P < 0.001). Women who thought that HDM offered health advantages (46.54%) were more likely to donate and receive HDM (P < 0.001).	Awareness and acceptability of HMBs are poor, but the majority had positive attitudes toward HDM. Targeted educational campaigns to promote HMBs are required.
Tian C et al. (2021) [15]	A brief, self-reporting, prospective, cross-sectional, online survey	To explore the level of, and the factors influencing, knowledge and attitude about donor milk among currently lactating women	489	Lactating women aged >18 years	HMB and DHM	40.1% of participants were aware of donor milk or milk banks, while 76.7% were willing to donate their milk. Various factors, such as parity, delivery mode, milk production, history of preterm delivery, education from health professionals, and infant age, influenced their attitude (P < 0.001).	The participants had positive attitudes toward donor milk. Nevertheless, there were knowledge gaps regarding donor milk.
Golsanamloo S et al. (2021) [16]	Cross-sectional analytical study	To identify knowledge and attitudes about donated milk and socio-demographic predictors of the health providers	535	Healthcare providers (272 nurses and midwives and 263 healthcare providers)	HDM	The general linear model showed that predictors of knowledge were having prior experience of breastfeeding another infant (P = 0.006) and encouraging others to breastfeed (P = 0.008). The predictor of attitude was aligned with encouraging others to breastfeed (P < 0.001).	Healthcare knowledge and attitude had a moderate impact on breast milk donation.
Huang C et al. (2021) [17]	Convenient sampling method	To understand the knowledge and attitude of breast milk donation among hospitalized mothers and provide data to support the establishment and development of breast milk banks in China	200	Hospitalized mothers	DHM	The overall correct answer rate about the knowledge of breast milk donation was not high (29.23%). The average score of breast milk donation attitude was relatively low, with a score of 32.97 ± 4.30.	The scope and intensity of breast milk donation are suggested to be expanded to promote the construction of breast milk banks.
Varer Akpinar C et al. (2022) [18]	Cross-sectional, mixed-methods study	To determine the opinions, knowledge, and attitudes of native Turkish and refugee women living in rural areas in Turkey about HMB	271	Women living in rural areas in Turkey	HMB	57.9% of women were open to donating breast milk, while only 27.7% were willing to use donor milk for their infants. The women expressed negative attitudes due to religious concerns, fear of diseases, and distrust of unfamiliar individuals.	Religious and cultural beliefs and safety concerns influence attitudes toward HMB.
Jahan Y et al. (2022) [19]	Prospective, cross-sectional study	To determine the opinions and attitudes among possible donor mothers regarding HMBs in one rural region in Bangladesh	121	Mothers aged 20-49 years, with at least one child, who was currently lactating or had breastfed her child, and who resided in the rural community of Bangladesh	HMB	Before talking with researchers, 98.3% of participants had never heard of HMBs. While the majority of participants (71.9%) would use human milk from milk banks if needed, 28% of moms said they would not use milk from a milk bank for their children. Of those surveyed, 99.2% were unaware that human milk banking methods are accepted in Bangladesh, and only 8.3% stated that HMBs were inappropriate in accordance with Islamic law.	A framework can be established for religious concerns regarding donors and recipients. It is recommended that health education by healthcare personnel and religious leaders can strengthen belief and increase awareness about milk banking practices among family members.
Özdemir S et al. (2022) [20]	Prospective cross-sectional descriptive study	To determine the knowledge and opinions of Turkish women in regard to donor milk banking and to raise awareness of donor milk banking	648	Female	HMB and DHM	54.1% of respondents were nulliparous, and 54.2% were aware of milk banks. 56.4% desired the establishment of milk banks in Turkey, and 50.8% considered donating milk. Reasons for rejecting milk banks included fear of disease transmission and potential incestuous relationships.	It appears that Turkish women lack information about donor milk banking. We recommend organizing public awareness activities concerning donor milk banking.
Tu H et al. (2022) [21]	Cross-sectional study	To understand women's acceptance of human milk banking in Wenzhou, southeastern China	305	Postpartum women	HMB	Only 17% of participants knew about milk banking. Many participants were willing to donate milk and use donor milk. Employment and knowledge of milk banking were predictors of willingness to donate milk. Monthly income, awareness, and knowledge of milk banking were associated with willingness to use donor milk. A majority of postnatal mothers had poor knowledge of milk banking. There was a significant association between demographic variables and knowledge of milk banking. No association was found between education, occupation, family type, and previous information with knowledge of milk banking.	The awareness of HMBs among women in the first year postpartum was low in this study. Mothers were more willing to donate human milk than to use donor human milk to feed their children. Knowledge of human milk banking was a predictor of both willingness to donate human milk and willingness to use donor human milk in this study.
Chauhan A et al. (2022) [22]	Descriptive non-experimental design	To assess the knowledge and attitude on human breast milk banking among postnatal mothers	100	Postnatal mothers	HMB	The participants had limited knowledge about human milk banking. A large percentage of postnatal mothers had average knowledge, while a smaller percentage had poor knowledge. Additionally, a majority of postnatal mothers displayed an unfavorable attitude. The study also found a significant association between demographic variables and the level of knowledge of human milk banking. However, no significant association was observed between education, occupation, type of family, or previous information and the level of knowledge on human milk banking.	The majority of postnatal mothers lacked knowledge and had negative attitudes toward human milk banking. Therefore, it is crucial for healthcare professionals, particularly nurses, to promote continued breastfeeding and raise awareness about human milk banking.
Kaur P et al. (2022) [23]	Descriptive study	To assess the knowledge regarding Human Milk Banking among mothers	60	Mothers	HMB	It was discovered that 48% of mothers had average knowledge, 40% had bad knowledge, and only 12% had strong knowledge. Mothers' knowledge and sociodemographic characteristics revealed a statistically significant link with the mother's educational status. The relationship between mothers' knowledge level and age, religion, source of knowledge, and involvement was not statistically significant (P < 0.05), but there was a statistically significant relationship between mothers' knowledge level and family income, education, and occupation.	The mother's educational status was significant in relation to knowledge. Age, religion, source of knowledge, and participation were not significant, but education, occupation, and family income were.
Jegannathan S et al. (2023) [24]	Semi-structured interview and focused group discussion	To find knowledge gaps, perception myths, and practice concerns related to staff nurses of NICU and lactating mothers	53	16 staff nurses and 37 lactating mothers	DHM	The majority of mothers were pleased to give milk. Recipient mothers agreed to use DHM but had worries about donor mothers' health and hygiene. Nurses shared their opinions on expansion, including enhancing awareness and infrastructure.	Need to scale up the human milk banking practices in low-middle-income countries.

Limitations of the study

The use of an online self-administered questionnaire may introduce sampling bias, as only students with internet access and willingness to participate in an online survey would be included. This may exclude individuals who have different perspectives or limited access to technology. Respondents may provide answers that they believe are socially acceptable rather than reflecting their true attitudes or behaviors. The study's findings may be specific to the geographical location where it was conducted. Cultural, regional, or institutional factors could influence attitudes and knowledge, and the results may not be applicable to other settings.

## Conclusions

The underlying principle behind this endeavor is to disseminate awareness among the fledgling cohort of medical and paramedical scholars regarding the act of donating human milk. A dearth of knowledge pertaining to human milk banking is rampant among medical and paramedical students, yet the gravity of the circumstances remains concealed. Consequently, it is imperative to extensively educate all Indian communities about the concept of human milk banking to gain widespread acceptance. Although the attitudes and practices toward HMB appear satisfactory, deficiencies in the student's comprehension and awareness of these conditions were detected. Despite the availability of institutions that facilitate the storage and distribution of human milk, there exists a considerable demand for such services in countries like India. To initiate consciousness concerning human milk banking and its registration process, it is indispensable to evaluate students' familiarity with this subject and devise novel educational strategies.
